# Correlation Between the Ratio of Oxygen Saturation to Fraction of Inspired Oxygen and the Ratio of Partial Pressure of Oxygen to Fraction of Inspired Oxygen in Detection and Risk Stratification of Pediatric Acute Respiratory Distress Syndrome

**DOI:** 10.7759/cureus.18353

**Published:** 2021-09-28

**Authors:** Pooja D Lohano, Sadam H Baloch, Murtaza A Gowa, Syed J Raza, Lareb Soomro, Hira Nawaz

**Affiliations:** 1 Pediatric Medicine, National Institute of Child Health, Karachi, PAK; 2 Pediatric Critical Care, National Institute of Child Health, Karachi, PAK; 3 Pediatrics and Endocrinology, National Institute of Child Health, Karachi, PAK; 4 Pediatric Medicine, Civil Hospital, Hyderabad, PAK

**Keywords:** pediatric acute respiratory distress syndrome, pao2 /fio2, spo2/fio2, lung disease, oxygenation index, oxygen saturation index, pulse oximetry, picu

## Abstract

Objective: To measure the correlation between the ratio of oxygen saturation to fraction of inspired oxygen [SpO_2_/FiO_2_ (SF)] and the ratio of partial pressure of oxygen to fraction of inspired oxygen [PaO_2_/FiO_2_ (PF)] among children diagnosed with acute respiratory distress syndrome (ARDS).

Methodology: A cross-sectional study was conducted at the pediatric intensive care unit (PICU), National Institute of Child Health (NICH), Karachi, a tertiary care government hospital, from November 2020 to July 2021. One hundred twenty children (of either gender) having the age range of 2 months to 16 years, admitted to PICU with acute onset of respiratory distress, were included in the study. We measured SpO_2_, PaO_2_, FiO_2_ and calculated SF and PF ratios. SPSS (version 23) (Armonk, NY: IBM Corp) was used to analyze data, and the Spearmen's correlation test was applied to measure the relationship between SF and PF ratios.

Results: A total of 120 children were included, the mean age was 40.58±38.88 months and 67 (55.8%) were males. The mean FiO_2_ was 76.33%, the mean PaO_2_ and SpO_2_ were 100.35 mmHg and 94.37%, respectively. The mean PF ratio was 156.34, and the mean SF ratio was 156.45. There was a strong correlation between the SF ratio and the PF ratio (r=0.688; p=0.001).

Conclusion: This study has shown that there is a strong correlation between the SF and PF ratios, and a statistically substantial agreement has been observed.

## Introduction

Acute respiratory distress syndrome (ARDS) is the most frequent clinical condition causing significant morbidity and mortality among children, especially, in the world's poorest countries [1]. In children, the incidence of ARDS is estimated to be 2.2 to 12.8 per 100,000 patients annually and 2.2% to 2.6% require pediatric intensive care unit (PICU) admission [2]. A recent report, including 145 worldwide ICUs from 27 countries, showed pediatric acute respiratory distress syndrome (pARDS) affected nearly 3% of PICU patients and 6% required mechanical ventilation [1]. However, the country-wise mortality rate of pARDS has varied largely from 20% to 75%, depending on the health care systems and their case-mix, income disparities, or a variety of other factors including geographic location, socioeconomic, and cultural differences [3-6].

The American-European Consensus Conference (AECC) in 1994 defined adult criteria of acute onset hypoxemia, which was used to characterize ARDS or acute lung injury (ALI) in children [7]. According to these criteria, the ratio of partial pressure of oxygen to fraction of inspired oxygen [PaO_2_/FiO_2_ (PF)] was used to define the hypoxemia. When the PF value was between 200 and 300 mmHg, the patient was diagnosed with ALI; however, when the PF value was less than or equal to 200 mmHg, the patient was diagnosed with ARDS [7]. Based on the severity of oxygenation compromise, the subsequent 2012 Berlin definition eliminated the taxonomy of ALI and classified ARDS into severe (PF ratio<100 mmHg), moderate (PF ratio ≥100 to ≤200 mmHg), and mild (PF ratio >200 to <300 mmHg) categories [8]. However, neither the AECC nor the Berlin criteria took into consideration the variations in etiologies, risk factors, pathophysiology, or outcomes between children and adults.

Recognizing that ARDS in children differs from that in adults, the Pediatric Acute Lung Injury Consensus Conference (PALICC) brought together an international panel of specialists to develop new definitions and recommendations for pARDS. The 2015 PALICC definition expands the radiographic requirement to encompass any new parenchymal infiltrates [9]. The new definition of pARDS also allowed the use of pulse oximetry, to prevent underestimating the incidence of ARDS in children when arterial blood gas data are not available. As a result, some pediatricians prefer to measure hypoxemia using the oxygenation index (OI) and oxygenation saturation index (OSI) instead of the PF ratio [9].

Evidence suggests that those diagnosed with ARDS based on their ratio of oxygen saturation to fraction of inspired oxygen [SpO_2_/FiO_2_ (SF)] have the same symptoms and outcomes as people diagnosed with PaO_2_/FiO_2_ (PF) and that having a consistently high oxygenation saturation index (OSI) at 24 hours is linked to poor pARDS outcomes [10]. Regular arterial blood collection to calculate the PF ratio is an invasive procedure and also has the risk of anemia and bleeding, especially in critically ill children. Pulse oximetry, on the other hand, is the most widely used, noninvasive, cost-effective, and safe method of monitoring oxygenation. Therefore, the SF ratio might be a desirable substitute in earlier diagnosis, severity assessment, and commencement of treatment in pARDS patients, particularly in resource-limited settings. There are no previous data available in this regard from Pakistan, and this research can provide the baseline information. Hence, this study aimed to measure the correlation between SF and PF ratios in critically ill pediatric patients with ARDS and to assess whether SF ratio could be used as a noninvasive alternative in risk assessment, diagnosis, and severity stratification or not.

## Materials and methods

It was a cross-sectional study, conducted at the PICU of the National Institute of Child Health (NICH), Karachi, a tertiary care government hospital, for eight months from November 2020 to July 2021. Using the WHO sample size calculator, considering the correlation between SF and PF ratios as 0.47 [10], power of test as 90%, and taking 99% confidence level, the estimated sample size was 60. However, we included 120 participants to increase the adequacy of the results. The children (of either gender) having the age range of 2 months to 16 years, admitted to PICU with acute onset (within seven days of known clinical insult) of respiratory distress, bilateral infiltrates on chest radiographs (consistent with the acute parenchymal disease), and presence of generalized edema (not fully explained by cardiac failure or fluid overload), were included in the study, whereas patients with chronic lung disease (e.g., pulmonary tuberculosis, asthma, cystic fibrosis) and congenital heart diseases were excluded from the study. Nonrandom consecutive sampling method was applied for sample selection.

This study was conducted after getting approval from the Institutional Ethical Review Board (IERB) of NICH (IERB No. 34/2020). Informed consent was taken from the parents/guardians of the eligible children, and the data were collected on predesigned pro forma. At the time of admission, demographic data such as the child's age, gender, weight, and oxygenation status were obtained. Shortness of breath, blue staining of the skin, and mucous membranes were all checked. The number of breaths per minute was used to calculate the respiratory rate. Parents and guardians were questioned about the start of symptoms. A chest X-ray was taken with an anteroposterior view and examined by a radiologist for the existence of infiltrates. With the probe affixed and the monitor linked, oxygen assistance was provided by face mask, nasal cannula, continuous positive airway pressure (CPAP), or mechanical breathing, depending on severity, and arterial blood gas was collected. At the same time, oxygenation parameters were also being measured.

All data were entered and analyzed using SPSS version 23 (Armonk, NY: IBM Corp). Mean±standard deviation was calculated for age, the weight of the child, and oxygenation parameters. Frequency and percentages were calculated for gender, presenting complaints, and the ARDS severity. Correlation between SF and PF ratios was determined using Spearmen’s correlation coefficient as they were not normally distributed. Agreement between SF and PF ratios for risk groups on the basis of Berlin definition was calculated using Kappa statistics. A p-value of ≤0.05 was taken as statistically significant.

## Results

A total of 120 children were included, the mean age was 40.58 months (range: 2-144 months), the mean weight was 12.27 kg (range: 2.3-40 kg), and they were predominantly males 67 (55.8%). The most frequent presenting complaint was fever (86.7%), followed by cough (30.8%). Of 120 children, 14 patients with ARDS required noninvasive ventilation (11.7%), and 106 patients required invasive mechanical ventilation (88.3%). The baseline characteristics of the study participants are displayed in Table 1.

**Table 1:** The baseline characteristics of the study participants (n=120) CPAP: continuous positive airway pressure.

Baseline features	Mean±SD
Age in months	40.58±38.88 (range: 2-144 months)
Weight in kg	12.27±7.97 (range: 2.3-40 kg)
	n (%)
Gender	
Female	53 (44.2)
Male	67 (55.8)
Presenting complaint	
Fever	104 (86.7)
Cough	37 (30.8)
Loose motion	18 (15)
Vomiting	16 (13.3)
Abdominal pain	8 (6.7)
History of burns	6 (5)
History of trauma	1 (0.8)
Supportive modality	
Noninvasive ventilation (CPAP, high flow)	14 (11.7)
Invasive mechanical ventilation	106 (88.3)

**Figure 1:**
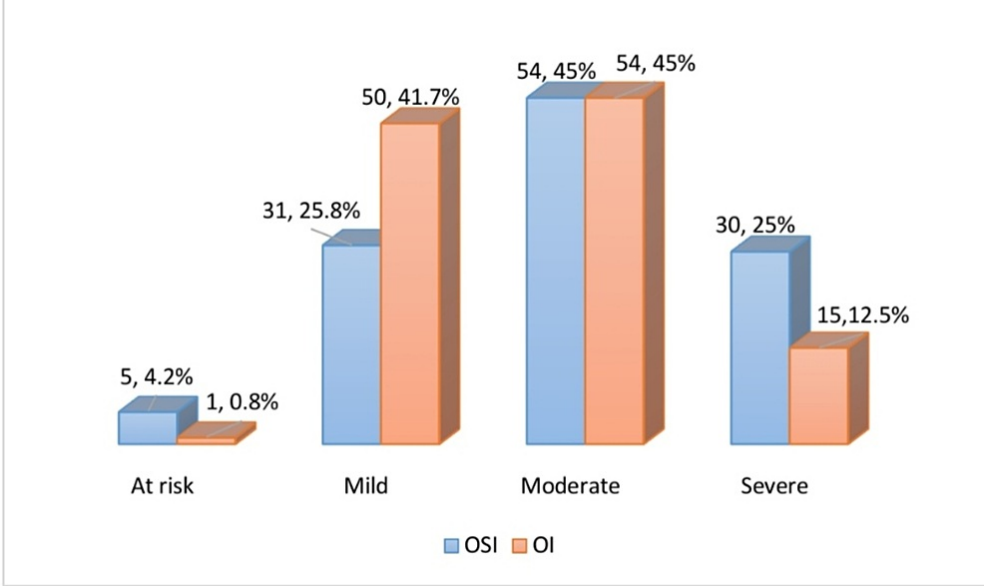
Frequency distribution of severity of ARDS ARDS: acute respiratory distress syndrome, OSI: oxygen saturation index, OI: oxygenation index.

The mean OI and OSI of all children were estimated as 10.19±5.37 and 10.08±4.41. According to the severity of ARDS based on OI, only one patient was at risk (OI<4.0), 50 patients had mild ARDS (OI: 4.0-7.9), 54 patients had moderate ARDS (OI: 8.0-15.9), and 15 patients had severe ARDS (OI≥16). While, according to OSI risk stratification, five patients were at risk (OSI<5.0), 31 patients had mild ARDS (OSI: 5.0-7.4), 54 patients had moderate ARDS (OSI: 7.5-12.2), and 30 patients had severe ARDS (OSI≥12.3) (Figure 1).

The mean FiO_2_ of children was 76.33%. Their mean PaO_2_ and SpO_2_ were 100.35 mmHg and 94.37%, respectively. The mean PaO_2_/FiO_2_ ratio was 156.34 (interquartile range [IQR]: 96.66-177.50), and the mean SpO_2_/FiO_2_ ratio was 156.45 (IQR: 97-165.83) (Table 2).

**Table 2:** Descriptive analysis of oxygen parameters measured FiO2: fraction of inspired oxygen, PaO2: partial pressure of oxygen, SpO2: oxygen saturation, PF: ratio of partial pressure of oxygen to fraction of inspired oxygen, IQR: interquartile range, SF: ratio of oxygen saturation to fraction of inspired oxygen.

Parameters	Mean±SD (range)
FiO2%	76.33±23.68 (10-100)
PaO2 mmHg	100.35±36.58 (36.40-237.0)
SPO2 %	94.37±5.32 (64-100)
PF ratio	156.34±117.05 (IQR: 96.66-177.50)
SF ratio	156.45±148.40 (IQR: 97-165.83)

**Figure 2:**
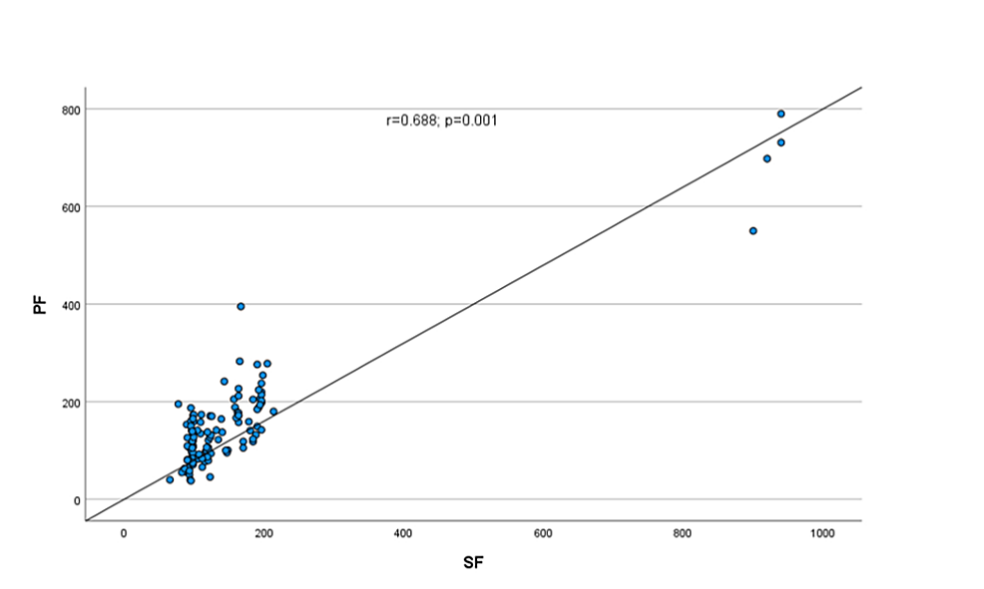
Correlation between SF and PF ratios among the study participants SF: ratio of oxygen saturation to fraction of inspired oxygen; PF: ratio of partial pressure of oxygen to fraction of inspired oxygen.

Since SF and PF ratios were nonparametric, therefore, Spearmen’s correlation was used to check the relationship between SF and PF ratios. There was a strong correlation between the SF ratio and the PF ratio (r=0.688; p=0.001) (Figure 2).

Furthermore, SF and PF ratios were categorized into two groups: at risk (PF≤300 and SF≤264) and not at risk (PF>300 and SF>264) on the basis of the Berlin definition. Of 120 children, 112 children were classified as at risk according to both SF and PF ratios, while five children were classified as not at risk or no longer need mechanical ventilation by both ratios. There was a statistically substantial agreement between SF and PF ratios (Kappa=0.75, p=0.0001) (Table 3).

**Table 3:** Agreement between SF and PF ratio for the risk stratification of pARDS pARDS: pediatric acute respiratory distress syndrome, SF: ratio of oxygen saturation to fraction of inspired oxygen; PF: ratio of partial pressure of oxygen to fraction of inspired oxygen.

PF	SF		Kappa-statistics	p-value	
	At risk	No risk			
			0.757	0.0001	
At risk	112 (97.4%)	3 (2.6%)			
No risk	0	5 (100%)			

## Discussion

ARDS is a major contributor to morbidity and mortality among children admitted to PICU. It has sophisticated diagnostic criteria that require an invasive blood gas analysis [2,11,12]. Concerns about anemia as a result of repeated blood drawing, as well as a desire to use less incursive methods, have led to fewer blood gas tests in critically ill patients. In this situation, the noninvasive and safe pulse oximetry saturation SF ratio could be utilized to diagnose and estimate the risk of pARDS [13-16]. Hence, in the current study, we have assessed the correlation between the PF and SF ratios for the diagnosis and risk assessment of ARDS in the pediatric population.

In our study, the mean age of the children was estimated as 40.58 months (approximately 3.4 years) and most of them were males (55.8%). Almost the same findings were observed in the study by Khemani et al., the sample's mean age was reported as three years, with the majority of the male children [17]. A Pakistani study by Ahmed et al. also revealed the mean age of the children with ARDS as 3.2 years and 61.3% of the males [18]. In the current study, in 120 suspected children who met inclusion criteria for the ARDS, the most frequent clinical complaints were fever (86.7%) and cough (30.8%). Singh et al. also found that fever was the most frequent clinical feature among 115 children with ARDS [19]. Similarly, Singhi and Mathew concluded that fever and cough were frequently seen clinical features in patients with respiratory distress [20].

In the present study, we found a strong correlation between SF and PF ratios (r=0.688; p=0.001) among 120 children with ARDS. In a similar study by Laila et al., a positive but weak correlation was observed between SF and PF among 39 patients with pARDS (r=0.215, p=0.189) [21]. In another similar study by Khemani et al., a moderate correlation was found between SF and PF (r=0.47) among 383 children with ARDS [10]. The difference in the correlation values might be due to the variations in sample size.

Without the use of blood gas analysis, SpO_2_ measurement with pulse oximetry might be a better technique for predicting PaO_2_ value. However, factors such as the placement of the oximetry sensor, race, methemoglobin, and underlying condition might reduce the diagnostic accuracy of the SpO_2_ measurement [21]. In the current study, we found the mean values of SpO_2_ and PaO_2_ as 94.4% and 100.35 mmHg, respectively. A multicenter study of 137 children conducted by Khemani et al. showed the mean SpO_2_ and PaO_2_ values as 95% and 70 mmHg [17]. Another study by Khemani et al. revealed the mean values of PaO_2_ and SpO_2_ as 69 mmHg and 94%, respectively [10].

On the basis of the Berlin criteria [22], we divided SF and PF ratios into two groups: at risk (PF≤300 and SF≤264) and not at risk (PF>300 and SF>264) in the current study. We discovered that SF and PF ratios were in good agreement. When it comes to risk stratification for pARDS, it is important to figure out which PF ratio matches best to the SF ratio. Hence, SpO_2_ can provide a viable noninvasive alternative for early detection of ARDS in children. Furthermore, it can also help in risk assessment and determination of the severity of lung disease, with the added benefit of avoiding arterial blood collection required for the measurement of PaO_2_.

There are certain limitations to this study. Although all the calculations were done simultaneously, the conditions that might shift oxygen-hemoglobin dissociation curve were not taken into consideration, and some of the PaO_2_ values were greater than 100 mmHg, and the SpO_2_ values were between the 64% and 100% range. Also, sample size is small, and all data were collected from only one PICU, and further multicenter researches with larger sample size are required in future, to increase accuracy of results.

## Conclusions

This study has shown that there is a strong correlation between the SF and PF ratios, and a statistically substantial agreement has been observed. So, the SF ratio can be reliably used for early detection and risk assessment of ARDS in children. 
